# Risk assessment of pollen allergy in urban environments

**DOI:** 10.1038/s41598-022-24819-w

**Published:** 2022-12-06

**Authors:** Talib Dbouk, Nicolas Visez, Samer Ali, Isam Shahrour, Dimitris Drikakis

**Affiliations:** 1 CORIA, UMR 6614, CNRS, Normandy University, UNIROUEN, 76000 Rouen, France; 2 Université de Lille, CNRS, UMR 8516-LASIRE-Laboratoire de Spectroscopie pour les Interactions, la Réactivité et l’Environnement, 59000 Lille, France; 3 Université de Lille, Institut Mines-Télécom, Université d’Artois, Junia, ULR 4515-LGCgE, Laboratoire de Génie Civil et géo-Environnement, 59000 Lille, France; 4 Laboratoire de Génie Civil et géo-Environnement, Lille University, 59000 Lille, France; 5 University of Nicosia, 2417 Nicosia, Cyprus

**Keywords:** Plant sciences, Health occupations, Medical research, Engineering, Mathematics and computing, Physics

## Abstract

According to WHO, by 2050, at least one person out of two will suffer from an allergy disorder resulting from the accelerating air pollution associated with toxic gas emissions and climate change. Airborne pollen, and associated allergies, are major public health topics during the pollination season, and their effects are further strengthened due to climate change. Therefore, assessing the airborne pollen allergy risk is essential for improving public health. This study presents a new computational fluid dynamics methodology for risk assessment of local airborne pollen transport in an urban environment. Specifically, we investigate the local airborne pollen transport from trees on a university campus in the north of France. We produce risk assessment maps for pollen allergy for five consecutive days during the pollination season. The proposed methodology could be extended to larger built-up areas for different weather conditions. The risk assessment maps may also be integrated with smart devices, thus leading to decision-aid tools to better guide and protect the public against airborne pollen allergy.

## Introduction

Digital engineering technologies based on advanced scientific methods could improve a city’s functionality, enhance its citizens’ well-being and facilitate economic growth. The COVID-19 pandemic revealed the need for developing and implementing risk assessment tools for hazardous airborne particulates. Examples include air pollution and airborne virus transmission under different weather or environmental conditions^1,2^.

According to projections made by the WHO (World Health Organization), by 2050, one in two people will suffer from an allergy disorder.

Moreover, with the last COVID-19 pandemic, additional hypotheses emerged regarding the relationship between airborne pollen exposure and airborne virus transmission^3^. Recent studies showed associations between allergy from airborne pollen and different viruses, including SARS-CoV-19^4–6^. For example, with evidence from 31 countries across the globe, Damialis et al.^4^ reported that higher airborne pollen concentrations correlate well with increased COVID-19 infection rates. Similar allergic diseases to COVID-19 relationships and effects were reported in different countries. For example, Yang et al.^6^ found evidence of an association between allergic diseases and the risk of adverse clinical outcomes of coronavirus disease. Gilles et al.^5^ addressed that pollen exposure weakens our innate defence against respiratory viruses.

Green zones close to urban environments, e.g., parks, plants, trees, and grass areas, are usually planted without considering pollination and airborne pollen transport under different weather conditions, buildings and transportation infrastructures. In other cases, the urban environment is created by removing green areas without prior attention to pollination from surrounding green zones.

Environmental conditions such as air pollution, toxic gas emissions, and climate change will modify the magnitude and timing of pollination^7^. Higher temperatures by the end of this century will displace the start of emissions by days or weeks for different types of pollen. Thus, pollen release seasons have been shifting earlier than usual, and their overall duration has been extending. Ziska et al.^8^ showed that the ongoing increase in global temperatures $T_{min}$ and $T_{max}$ might already be contributing to extended seasonal duration and higher pollen loads.

**Figure 1:**
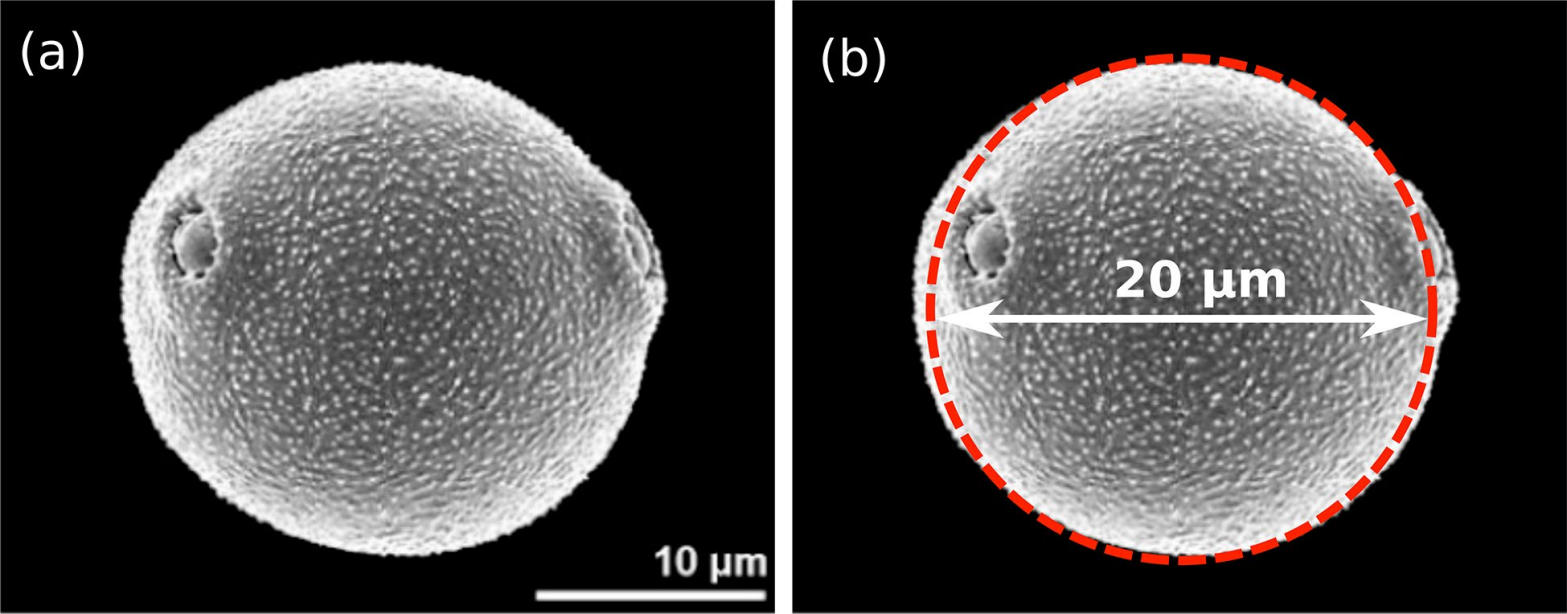
Pollen grain microscopy. (**a**) Birch (*Betula pendula*) pollen grain (source: Halbritter et al.^10^); (**b**) showing an approximated spherical shell of $20\,\upmu$m diameter (red color dashed-curve) used in the present computational model.

Birch (*Betula pendula*) is one of the most allergenic pollen-producing trees; over 100 million people are allergic to birch pollen grains^9^ (Fig. 1)^10^. Pollen-related allergic diseases have been rising for decades, and the reasons behind this increase are unknown^11^﻿. Still, environmental pollutants, such as diesel exhaust particles, appear to play a role^12^. In the last few decades, concentrations of airborne birch pollen were increased, and the duration of exposure was lengthened due to climate change^13^. With a size of about twenty micrometres^14^ and a mass of a few nanograms^15^, birch pollen encompasses a high potential for long-distance transport of up to thousands of kilometres^16,17^. Atmospheric concentrations of birch pollen can reach several thousand pollen grains per cubic meter. The amount of 45–70 pollen grains per m$^3$ is considered sufficient to trigger symptoms in allergic people^18,19^. However, the precise threshold beyond which allergic symptoms may occur is still debatable.

Therefore, knowledge of the atmospheric concentration of allergenic pollen grains is critical in preventing symptoms. Pollen grain sensors are usually installed on roofs at a height of 15–20 m, and the pollen concentrations may differ from ground level, where exposure mainly occurs^20^. Ground-level measurements in urban areas show high variability in local and spatial pollen concentrations^21^. On a regional scale, pollen grain concentrations can be spatially and temporally highly heterogeneous depending on the proximity of sources but also on the urban topography of the environment. In recent research, Sousa-Silva et al.^22^, following a “riskscape” concept, presented evidence on how tree pollen allergenicity datasets can shape the risk for pollen-allergy sufferers. Their study considered five cities with different urban forests and population densities.

As discussed above, pollen seasons have been shifting and extending. Therefore, higher pollen emissions will induce airborne pollen transport at a large scale in space^23^ and time^24^ that it would be challenging to quantify experimentally. This will make airborne pollen release a significant public health issue worldwide where its risk assessment in an inhabited urban environment will be vital. So, it will be crucial to accurately predict local airborne transport in urban environments, which is hard to achieve experimentally. Thus, the present study sheds light on the role of computational science in predicting the local transport of airborne pollen at the scale of a University Campus of about 1.75 km$^2$ (50 m in height) as an intelligent city prototype. This will allow us, after live monitoring, to better design smart cities or urban infrastructures. Furthermore, by developing advanced risk assessment and decision-making tools, we could limit the exposure of allergic people to pollen at a street level.

We employ multi-physics and multi-scale computational modelling and simulations to investigate airborne pollen transport on a university campus in the north of France under different weather conditions. We compute the local transportation of about 0.4 million pollen grains emitted initially by the trees and transported by the wind towards the surrounding largest urban zones, e.g., buildings infrastructure, campus library and the subway metro line bridge.

The present study is limited to Birch (*Betula pendula*) pollen^10^ but can be extended easily to other pollen, especially if the size and density of other pollen grains are comparable. The present work sheds light, for the first time in the literature, on the vital role of computational fluid dynamics (CFD) in the risk assessment of pollen dispersion/allergy in urban environments. Pollen production and emission are highly individualistic and thus depend largely on the sampling year, station, micro-locality, and tree topology. The CFD model developed in this paper constitutes a baseline version that can be extended further for pollen transport in the urban environment at larger scales. For example, it can consider the high-elevation transport phenomena of pollen across regions^25,26^. This is in addition to future simulations at extended airborne pollen grains transport periods.

## Results

We present results from different computational fluid dynamics (CFD) simulations regarding the pollen conditions at the university campus between the 6th and the 10th of April 2020.

We set $n_{max}$ as the initial total pollen grains emitted or produced by all the trees on the campus after an initial period $t=\delta \tau$ ($\tau \rightarrow ~0^+$). $\delta \tau$ corresponds to the period a pollen grain needs to naturally detach from its flower, e.g. after a wind impact that induces its release from flower-borne to airborne. Thus, this short period $\delta \tau$ is very natural and, in our model, was not imposed, but it is observed to be very small. It slightly varies from one pollen grain to another, but in general, for most pollen released on the campus at the corresponding weather conditions, we observe that $\delta \tau ~\le ~0.01$ s. $\delta \tau$ is physics-based and corresponds to a natural-like pollen tree-to-air detachment period. $N_{\delta \tau }$ is the initial total number emitted by one tree such that $n_{max}=n_{trees} \times N_{\delta \tau }$, where $n_{trees}$ is the total number of trees on campus ($\approx 1187$). We consider a mean pollen density^27^ of ${\rho _p}=800~\text {kg}\,\text {m}^{-3}$ and a mean pollen grain diameter of $d_p=~20\,\upmu$m (see Fig. 1).

**Figure 2:**
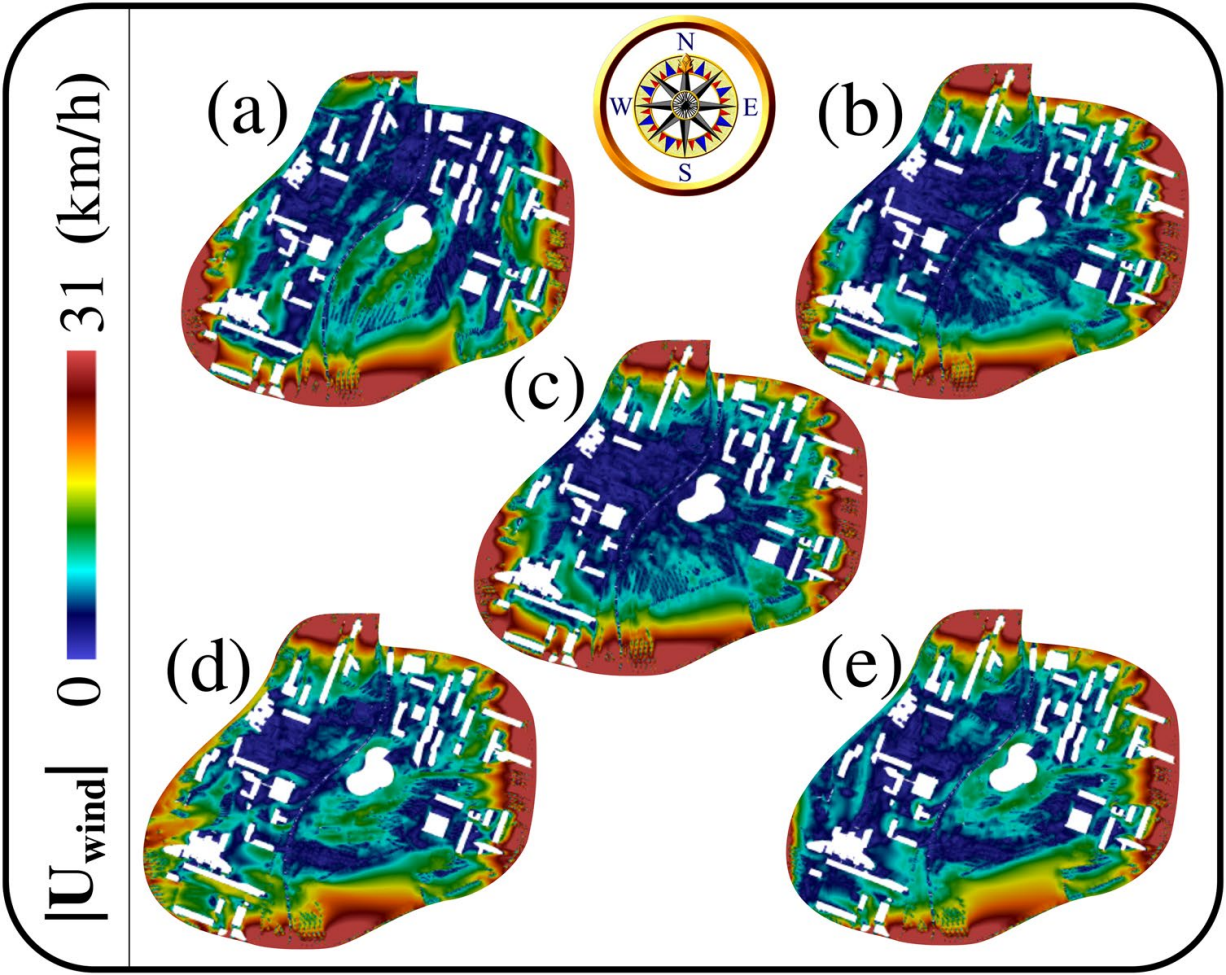
Local wind speed magnitude maps computed at $t=6$ min. In the centre, one can see the Library of the University of Lille and the Lilliad Learning Center Innovation^28^. Both are frequently visited by scientists and students from and outside the campus. CFD results at $z=5$ m above the ground level $z=0$; computational results for the 5 days: (**a**) 06/04/2020; (**b**) 07/04/2020; (**c**) 08/04/2020; (**d**) 09/04/2020; (**e**) 10/04/2020.

Figure 2 shows an example of the velocity magnitude field (or wind speed maps) obtained at $t=6$ min in the horizontal xy-plane at $z=5$ m. The urban infrastructure topology affects the wind speed locally inside the university campus. For example, as Fig. 2a–e show, the wind speed around the central building (university library) varies in the space at a steady-state of wind at $t=6$ min. Due to the infrastructure and weather conditions, these steady-state wind speed daily variations will induce steady local wind corridors between the different buildings. These wind corridors will thus orient the airborne pollen grains from the sources to preferred directions leading to their non-homogeneous dispersion on campus. Thus, the pollen grains will be convected to specific local regions depending on the weather conditions and the wind interaction with the urban infrastructures (see also “Discussion” later).

### Airborne pollen grains interaction with urban infrastructures

**Figure 3:**
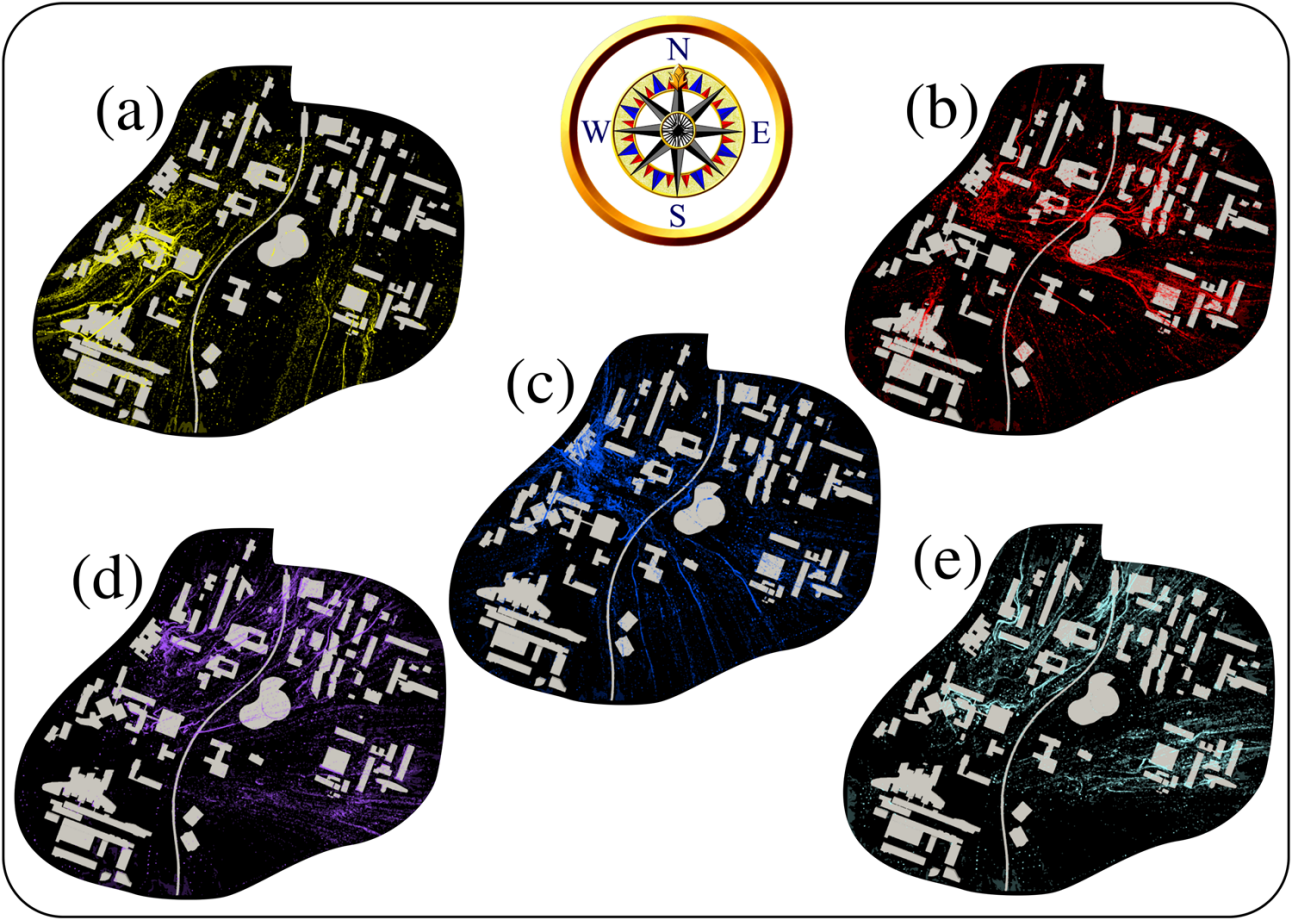
Effect of weather conditions and urban infrastructure on airborne pollen aerodynamics outdoors in a university campus. Computational results at $t=6$ min are shown from a top-view 3D perspective. (**a**) 06/04/2020 (yellow-colored grains); (**b**) 07/04/2020 (red-colored grains); (**c**) 08/04/2020 (blue-colored grains); (**d**) 09/04/2020 (magenta-colored grains); (**e**) 10/04/2020 (cyan-colored grains); see Table 1. The initial number of pollen grains, in this case, is $n_{max}\approx 0.4\times 10^6$. For the complete aerodynamics at all time steps, see the multimedia video files: Video 1, Video 2 and Video 3.

Wind corridors emerge with time and orientate a considerable amount of airborne pollen grains inside the university campus. This can be observed in Fig. 3 at $t=6~min.$ for five different weather data conditions (see Table 1). Figure 3a shows that many airborne pollen grains are locally concentrated in regions west of the university campus. This can be explained by the interaction between the pollen and weather conditions (on 6th April 2020) and the urban topology of the largest infrastructures (e.g. buildings, largest trees and subway metro line bridge). Furthermore, under different weather conditions on another date, e.g. Fig. 3d, different local zones in the campus witness high concentrations of airborne pollen grains (e.g. North and North-East zones).

One may question the effect of the total pollen production $n_{max}$ and density $\rho _p$ on the overall aerodynamics (e.g. settling, wind-infrastructures interaction) and the local pollen dispersion on the university campus.

To answer the above question, we conducted large-scale simulations using the 6th April 2020 weather conditions with ten times larger than the initial number of pollen grains emitted by the trees, thus increasing $n_{max}$ by tenfold.

**Figure 4:**
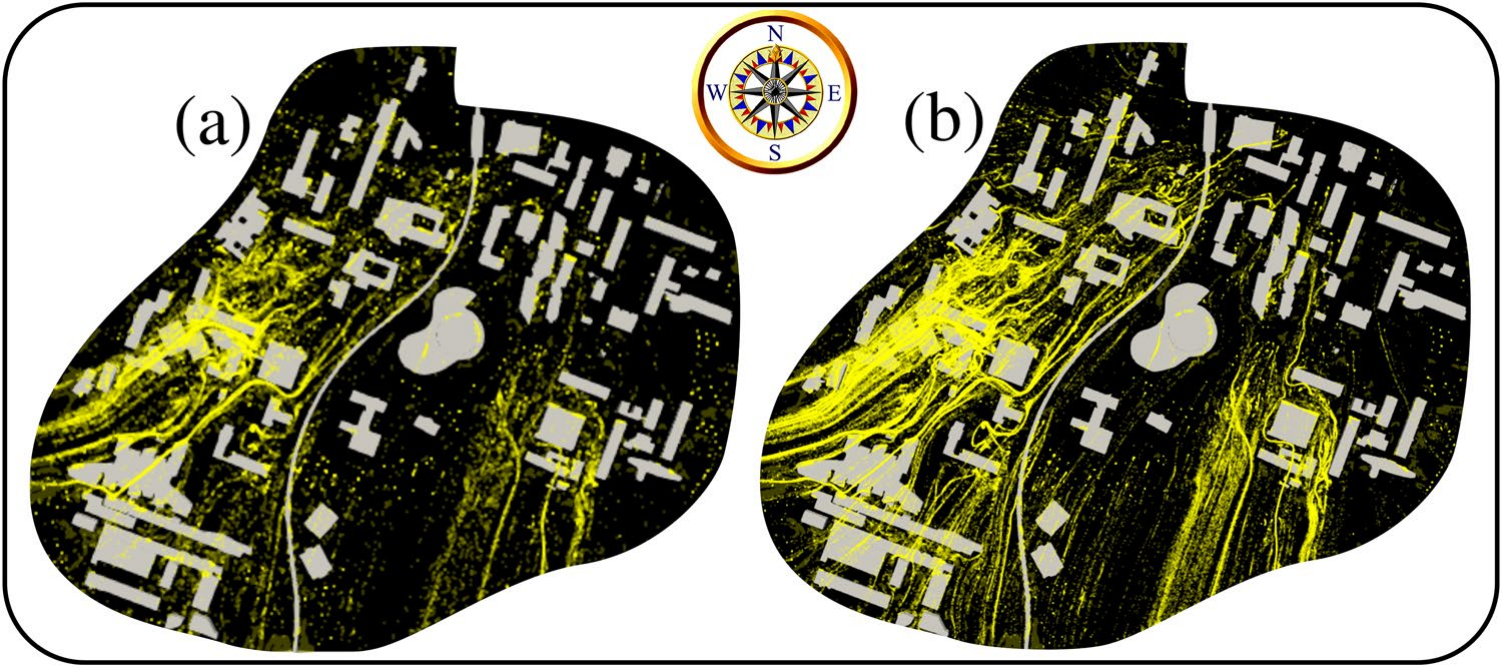
Effect of pollen grains total production or maximum number ($n_{max}$) imposed at $t=\delta \tau \rightarrow ~0^+$. The case is of the weather conditions on 06/04/2020. Computational results at $t=6$ min. (**a**) Domain top view with $n_{max}\approx 0.4 \times 10^6$ pollen grains; (**b**) domain top view with $n_{max}\approx 4 \times 10^6$ pollen grains.

**Figure 5:**
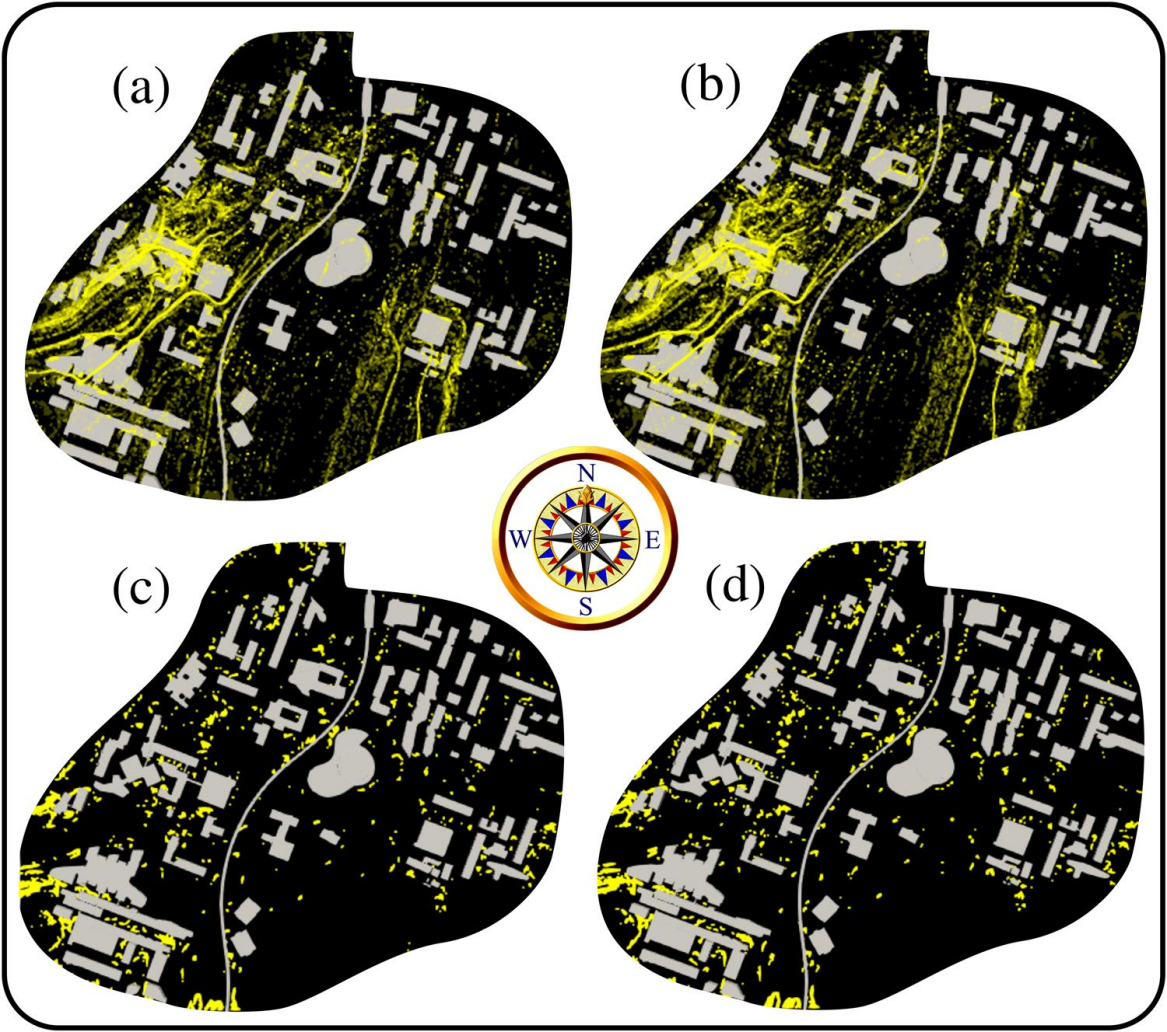
Pollen grain density effect investigated at the weather conditions on 06/04/2020. Computational results at $t=6$ min. (**a**) Domain top view with $\rho _p=800~\text {kg/m}^3$; (**b**) domain top view with $\rho _p=1700~\text {kg/m}^3$; (**c**) pollen grains at the ground level with $\rho _p=800~\text {kg/m}^3$; (**d**) pollen grains at the ground level with $\rho _p=1700~\text {kg/m}^3$.

The computational results in Fig. 4 show that increasing the number of initial pollen grains emitted by the trees at the instant $t=\delta \tau \rightarrow ~0^+$ does not significantly modify the local pollen grains corridors preferred by the wind and its interaction with the urban infrastructures. We then conducted a CFD simulation using the 06/04/2020 same weather conditions but using a different mean pollen grain density of 1700 kg/m${^3}$ (Fig. 5). From Fig. 5a,b, we observe that doubling the density value does not significantly affect the local aerodynamics of the pollen grains on the university campus. The above can be explained by the ratio of the aerodynamic force induced by the wind speed and the drag force due to settling. Nevertheless, Fig. 5c,d show that more pollen grains are settled to the ground at $t=6$ min.

## Discussion

### Quantitative analysis

Measuring airborne pollen spatially in high resolution is a challenging task. This is due to instrumentation constraints, expensive sensors and their installation in a dispersed, homogeneous environment across a city. Nevertheless, the proposed numerical approach for predicting airborne pollen transport on an urban scale provides a meaningful way forward. For example, using CFD modelling, we can enhance the quantification of pollen concentrations in targeted zones of a town. Furthermore, we can apply the methodology to any zone inside the computational domain to quantify the total number of pollen grains, their complex aerodynamic behaviour, and their interaction with the surrounding urban infrastructures, e.g., trees, buildings, and the subway.

**Figure 6:**
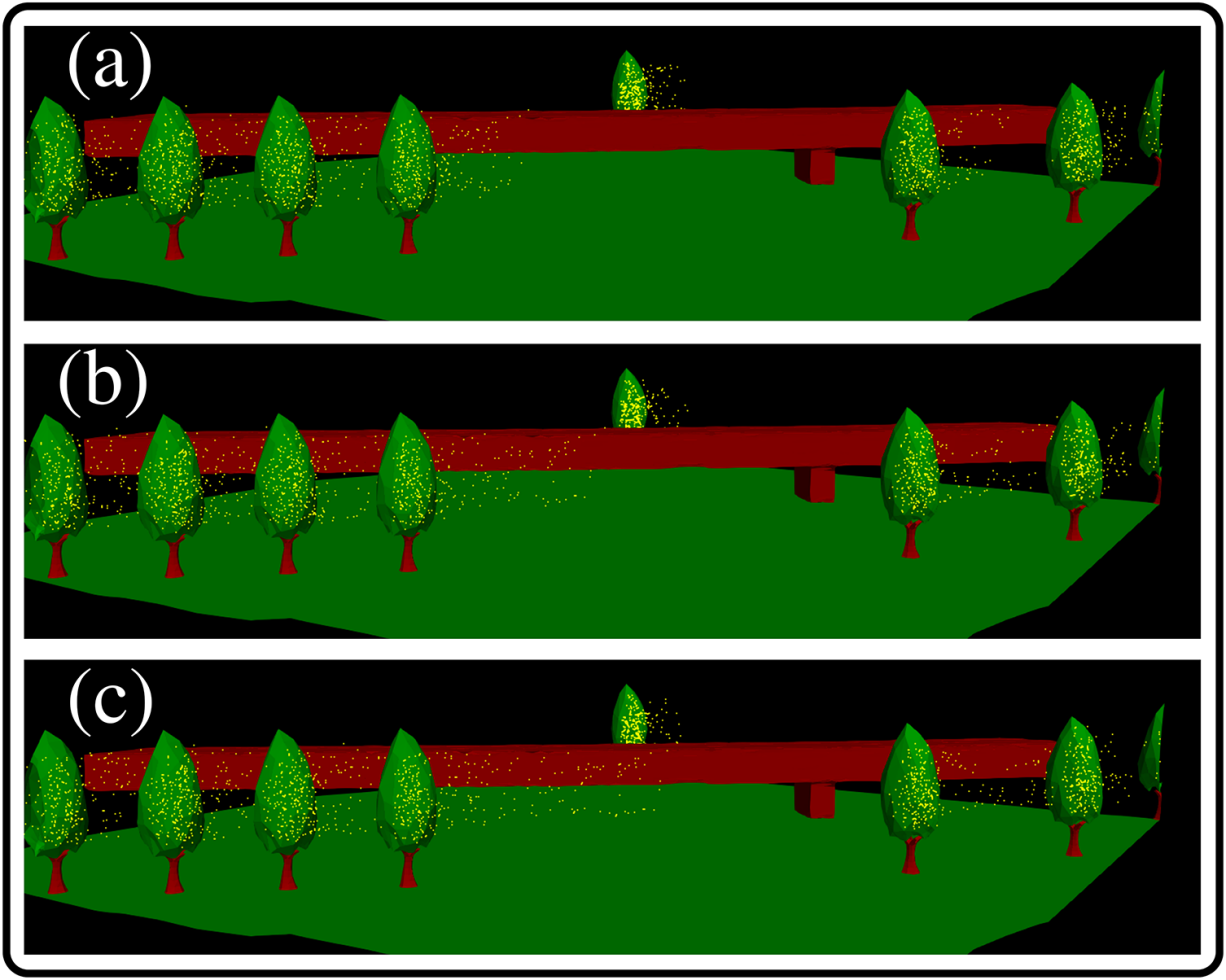
Pollen transport in a region **R** inside the university campus showing grains-trees detachment due to the wind-tree-leaves interactions. (**a**) t = 1 s; (**b**) t = 2 s; (**c**) t = 3 s. Computational case of the weather conditions on 06/04/2020 with a total initial number of pollen grains $n_{max}\approx 0.4\times 10^6$. A local region **R** of the whole domain corresponds to a box of dimensions: $L=W=100$ m and $H=40$ m (see “Methods” section). For the complete aerodynamics at all time steps, see the multimedia video file: Video 4.

Figure 6 illustrates an excellent example of the proposed method’s potential for investigating pollen dynamics in an urban environment. It sheds light on the importance of the space and time scales in accurately modelling local airborne dispersion. It is essential for investigating future sampling pollen technologies and pollen allergy risk assessment at large scales. The present modelling strategy (Fig. 6) takes into account different conditions, such as the weather, urban infrastructure, the ground topology and the various complex mechanical interactions, which can significantly affect airborne pollen dispersion and deposition.

Experimental data of pollen grains (PG) concentration in the air were recorded in a single location on the rooftop of one building on the campus. This experimental data was recorded in 2020 using a Hirst-type (Hirst^29^) pollen trap placed on a rooftop of a building situated on the North/West side of the University campus. This history data of pollen counts provided by the French Airborne Pollen National Surveillance Association^30^ show that the period 06/04/2020 to 10/04/2020 corresponds to the period of highest pollen grain average concentration in air^29,30^ of about 1160 $PG/{{\text {m}}^3}$ (as one average concentration value reported per day).

**Figure 7:**
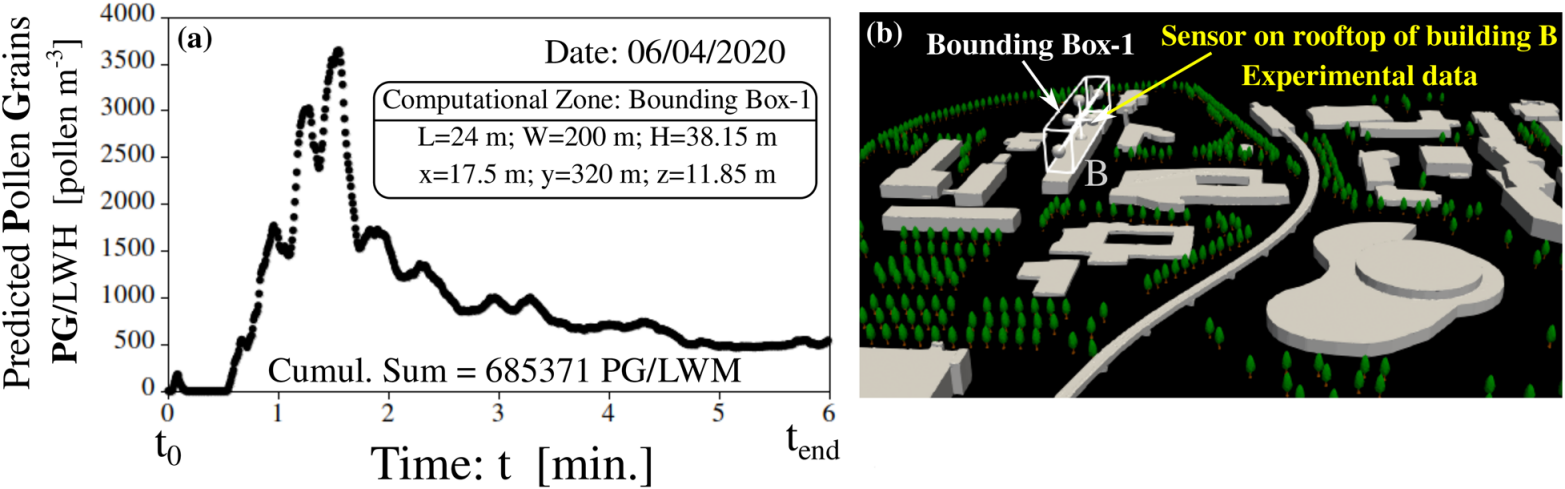
Numerically predicted pollen grains count over 6 minutes on the University campus in Lille-France. Numerical predictions of pollen grains (PG) as a function of time (**a**); computed for grains passed through a numerical acquisition Box-1 that mimics the experimental sensor located on the rooftop of building B (**b**). The dimensions of the Box-1 are $L=200~{\text {m}};~W=24~{\text {m}};~H=38.15~{\text {m}}$, where *L* denotes the length, *W* is the width, and *H* is the height of this Box-1.

We compared the high-spatial-resolution (HSR) numerical findings to the low-spatial-resolution (LSR) experimental data for the count of pollen grains (*PG*) measured in SI units in $PG/{\text {m}}^{3}$ using the following approach. We used the numerical results of Fig. 7a, for the case of $N_{\delta \tau } \approx 3200$ particles per tree, corresponding to the case of Fig. 4b at $n_{max}=4\times 10^6$ PG. In the same building-B (Fig. 7b), a sensor was placed on the rooftop^29^, located in our computational domain at $x=17.5~{\text {m}};~y=320~{\text {m}};~z=11.85~{\text {m}}$. Therefore, we defined a new data acquisition bounding Box-1 of dimensions $L=200~{\text {m}},~W=24~{\text {m}},~H=38.15~{\text {m}}$ (see Fig. 7a).

Box-1 allows one to compute the PG count as a function of time (Fig. 7) and to deduce an average cumulative sum of all the pollen grains that passed through this Box-1 during the total simulation period (e.g. in this case $=~6$ min). As Fig. 7a shows, the cumulative sum (TCS) at the end of the simulation is found to be 685,371 *PG*/*LWH*, where *LWH* denotes the volume of our Box-1 in ${\text {m}}^3$.

Substituting ($L=200~{\text {m}},~W=24~{\text {m}},~H=38.15~{\text {m}}$) in *LWH*, gives a final numerical value of about 40 $\text {PG}/{\text {m}}^3$. This difference is interesting and can be explained by the fact that airborne pollen sampling agencies usually try to minimize the local effects by placing the Hirst pollen sensors 20–30 m above the ground. However, these sensors are sparsely scattered in urban spaces. Therefore, the information provided by these rooftop sensors is likely to be relevant instead of mesoscale transport, e.g., see Rantio-Lehtimäki et al.^31^. Furthermore, the rooftop location of the Hirst sensor was chosen to preferentially catch pollen grains from outside the campus which we do not consider in our modelling. Finally, one should not forget that in Fig. 4, we showed that increasing the total pollen production in the campus ($n_{max}$) did not affect the steady local dispersion paths adopted by the pollen grains inside the campus. Further numerical and experimental research is still required to quantify the elevation effect better.

In future studies, we plan to install multiple physical sensors to measure PG count in different positions inside the University Campus, e.g., obtain experimental data at different elevations and a high-resolution spatial scale (numerous local positions). This will allow us to measure PG count in space and time accurately. We are also working on an advanced laser-based sensor that can fit nicely inside a tree to provide better information on the pollen grains emission rate ($\text {PG}/{\text {min}}$) per tree (or the parameter *Pr* detailed in “Methods” section). This will permit more quantitative experimental data to compare with simulation data. Finally, a more detailed parametric analysis is also planned to investigate the effect of the different numerical model parameters on the relative error.

Another future objective is to couple weather forecasting models with CFD models to better forecast pollen dispersion considering the built-in infrastructures present in the urban zone during periods of human activity.

### Risk assessment maps

**Figure 8:**
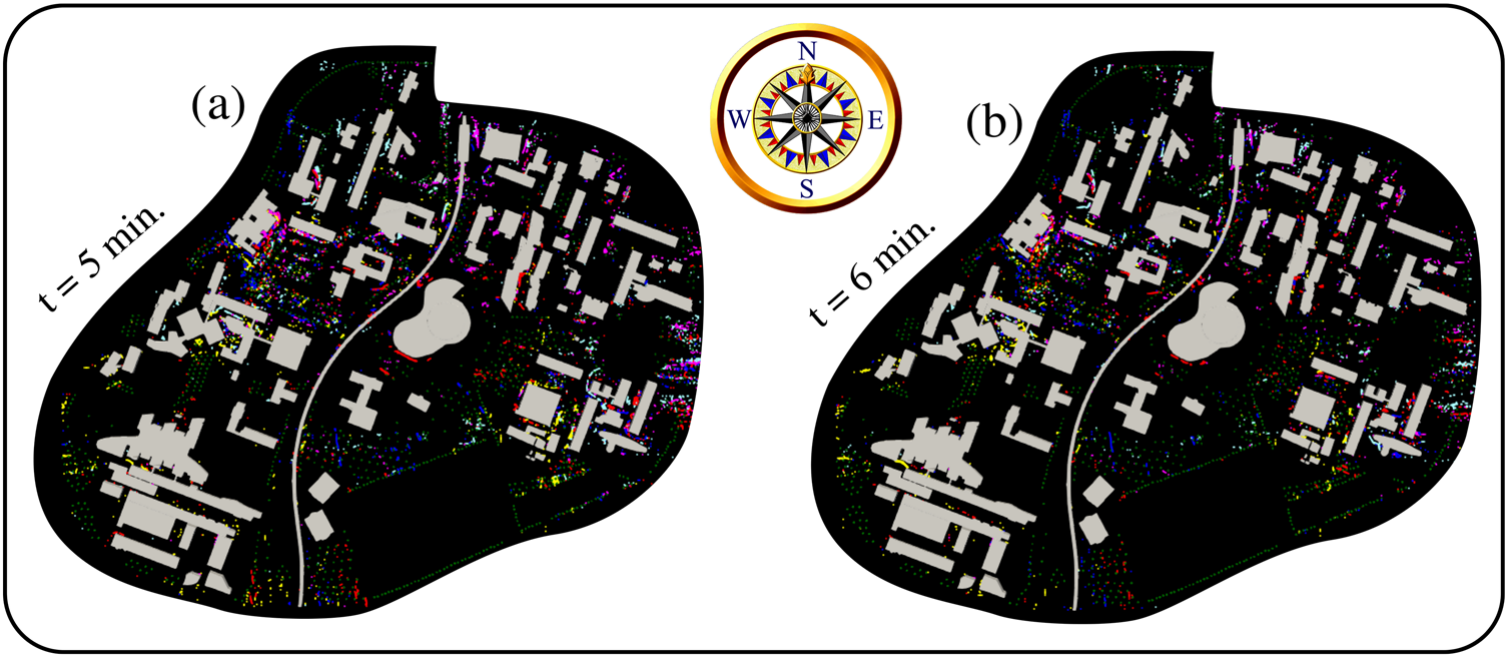
Risk assessment maps obtained from advanced large-scale computational fluid dynamics simulations, including the trees in green colour (initial sources of pollen emission). Pollen allergy zones inside a university campus. The five different colours correspond to pollen grains between 0.5 and 2.5 m above the ground level (a dangerous height interval where people stand and are more exposed). Yellow: 06/04/2020; red: 07/04/2020; blue: 08/04/2020; magenta: 09/04/2020; cyan: 10/04/2020. For the complete aerodynamics at all time steps, see the multimedia video file: Video 5.

We present below the methodology for constituting risk assessment detailed maps for airborne allergy based on CFD methods. Suppose we assume that most university students will have different heights. These people will likely inhale pollen grains between 0.5 and 2.5 m above the ground. Figure 8a,b show the superimposed results during five consecutive days of pollen grains between 0.5 and 2.5 m above the ground. The above allows the development of risk assessment maps for airborne allergy pollen in a city with complex urban infrastructures. Figure 8 represents the projection of local pollen grains positions (5–6 min) in the human-risk-subdomain (0.5 and 2.5 m). Figure 8a,b show the accumulation of pollen for 5 days from the first time instant $t=0$ when the pollen starts to detach from the flowers to the environment, thus becoming airborne after $t=\delta \tau \rightarrow ~0^+$. The future integration of this new methodology with artificially intelligent decision-aid tools in smart devices can better guide and protect the public against airborne pollen allergies.

In conclusion, we developed a multiscale computational fluid dynamics methodology for predicting the transport of airborne pollen grains within urban infrastructure for the first time. Several simulations have been conducted under different weather conditions to constitute risk assessment maps of airborne allergy pollen that can better guide and protect the public in pollination seasons. The weather data used in the CFD simulations were adopted from the historical data recorded by a weather station in the north of France. The airborne pollen grains’ transport, aerodynamic behaviour and their interaction with the urban infrastructures in a university campus have been quantified, analyzed and discussed. The proposed methodology provides new perspectives on predicting airborne pollen grains dynamics and concentrations in different local zones within a city, which is difficult to measure experimentally.

## Methods

### Computational modelling

**Figure 9:**
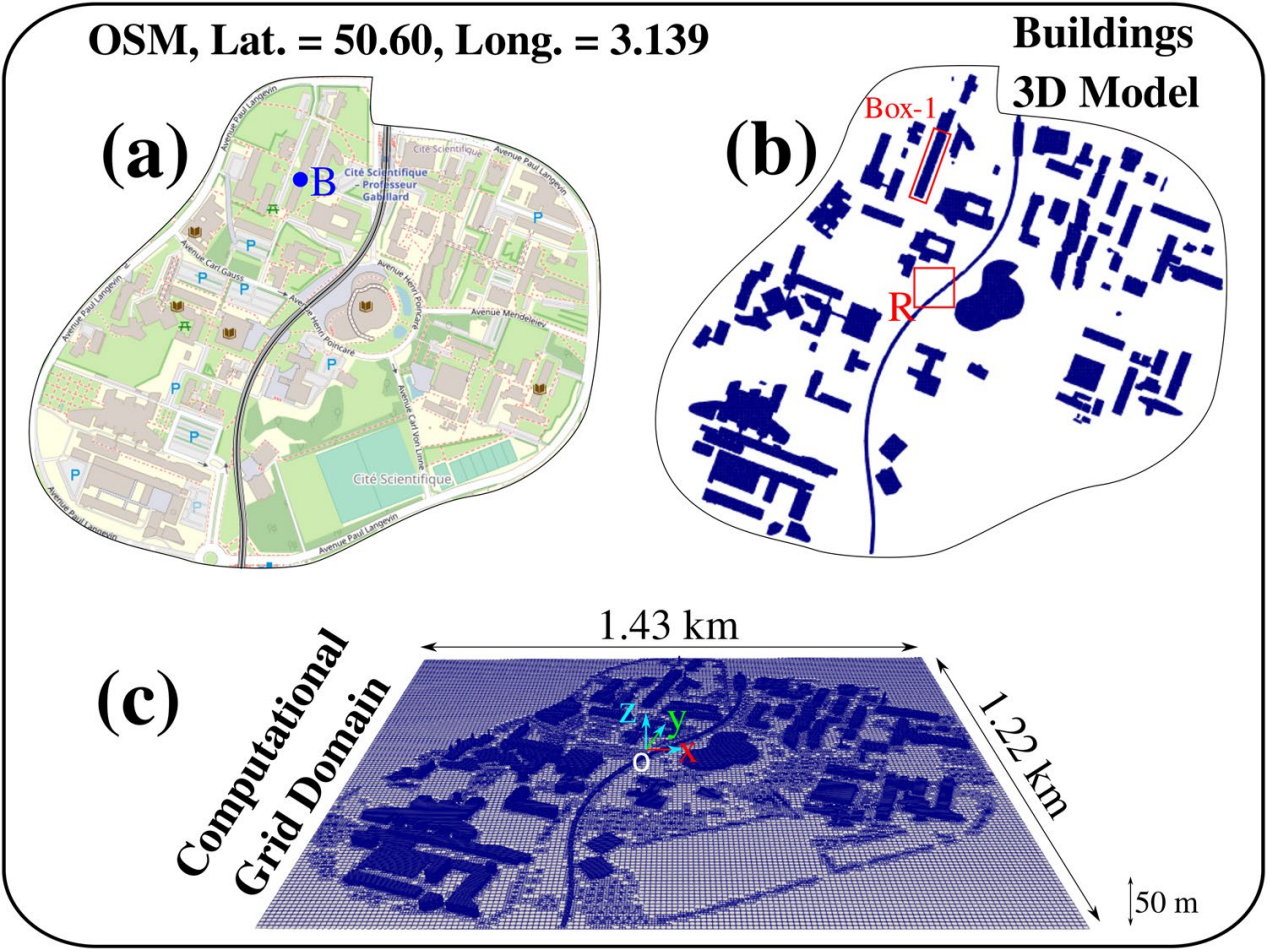
The Campus of the University of Lille at Villeneuve d’Ascq, F-59000, France. (**a**) Map of buildings with heights extracted from the buildings database of www.openstreetmap.fr (OSM) showing the local position of Hirst sensor^29^ on the rooftop of Building-B installed in by the French Airborne Pollen National Surveillance Association^30^; (**b**) the 3D constructed computer-aided design (CAD) model for the buildings and subway metro line urban infrastructure, showing two local regions for local pollen quantification post-processing and validation; Box-1 at $x=17.5~{\text {m}};~y=320~{\text {m}};~z=11.85~{\text {m}}$ with dimensions $L=200~{\text {m}},~W=24~{\text {m}},~H=38.15~{\text {m}}$; Region **R** at centre with dimension $L=W=100~{\text {m}},~H=40~{\text {m}}$; (**c**) an example of the computational grid and domain used to compute the airborne pollen transport in each cell inside the university campus of about $1.75~{\text {km}}^2$ surface area and up to 50 m above the ground level.

Figure 9 shows a 2D map, a buildings model and a computational 3D model of the campus of the University of Lille in *Villeneuve d’Ascq*, F-59000, France.

We model airborne pollen transport at the urban scale, focusing on the largest built-in structures and the largest trees as sources of airborne pollen grains. The model is based on the solution of the Navier–Stokes equations, which include both convection and diffusion. In addition, we solve the flow dynamics of each pollen grain, including all the physical forces (conservation equations of mass, momentum and energy). The modelling framework includes the local interactions between the wind, the pollen grains and all the structures in the computational domain. The detailed mathematical derivations of our computational model were presented in^1–3,32^.

**Figure 10:**
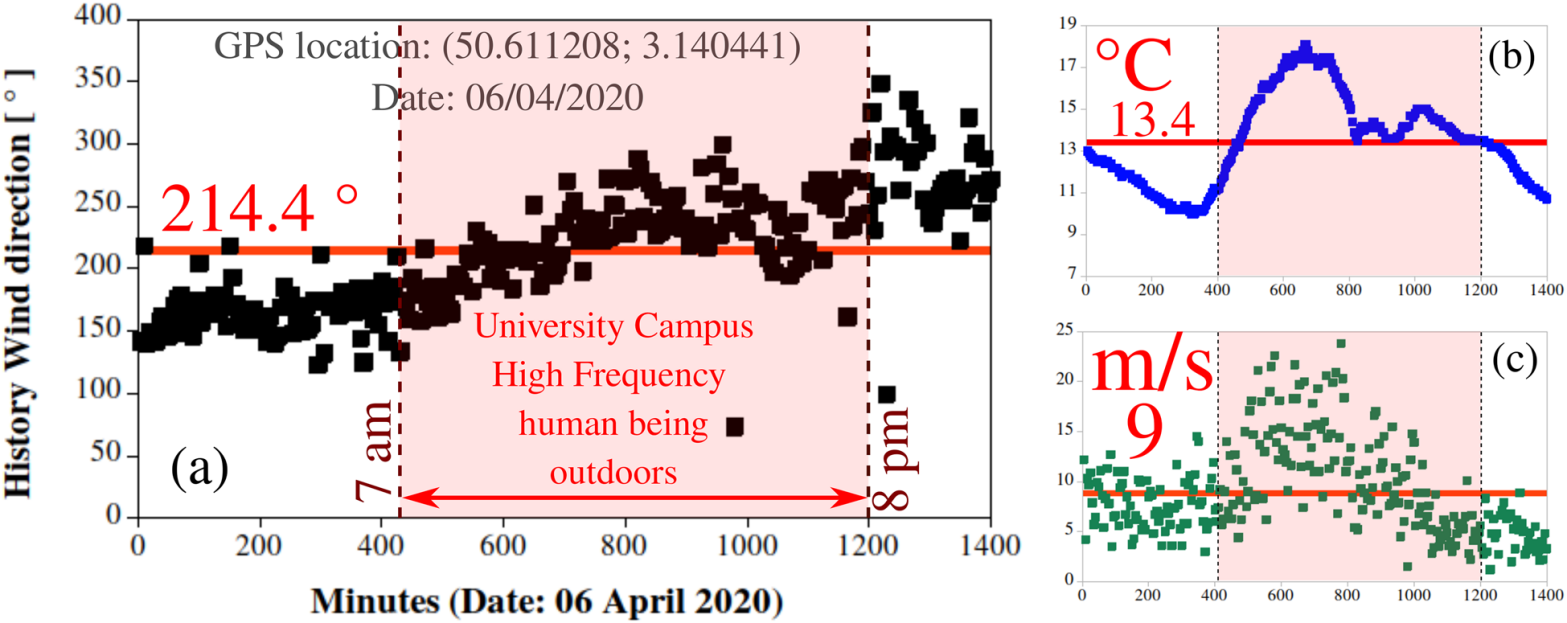
An example of history wind direction on 06/04/2020 recorded at every 5 min showing the average value and the period (7 am–8 pm) as high frequency of human being outdoors in the university campus. (**a**) Wind direction in $\circ$; (**b**) temperature in $^{\circ }$C; (**c**) wind speed in m/s. The weather station is located at the GPS position (50.611208; 3.140441) 60 meters above the sea level (masl); *manufacturer: RPG-Radiometer; model: RPG-HATPRO*.

The transport and conservation equations are discretized via the Finite Volume Method^33,34^ with second-order schemes. The RANS (Reynolds Averaged Navier Stokes) turbulence model $k-\omega -SST$ accounts for turbulence’s effect in the modelling. Mesh sensitivity analysis is conducted using three different mesh sizes (5, 8 and 15 M cells). The 5M cells were adopted based on a maximum 5% error in the GCI index^35^ (based on the local velocity field in a local zone at the scale of a building). Near-wall mesh refinements are applied such that turbulent wall functions are adequately used in near-wall zones with $50< y^{+} < 500$. The mesh topology is non-uniform hexahedra with mesh refinement in zones close to the buildings and built-in infrastructure, e.g., the minimum grid-cell size is about 1.13 cm for the adopted 5 million cells mesh. On the other hand, the maximum grid-cell size in open-air zones far away from the structures is 10 m. In environmental (open space) CFD simulations, the minimum mesh cell size can vary from a few meters to several kilometres. The environmental conditions in the computational domain (temperature, pressure, relative humidity, wind speed and direction) are all imposed to values corresponding to a natural spring season. Therefore, they indicate actual weather history data recorded at the university campus during five consecutive days between the 6th and 10th of April 2020. The weather station is located at the GPS position (50.611208; 3.140441), 60 m above sea level (masl); *manufacturer: RPG-Radiometer; model: RPG-HATPRO*. Despite their excellent time scale high resolution, one should bear in mind that this weather station provided relatively reduced spatial resolution. An example of weather history for the 6th of April 2020 is illustrated in Fig. 10, which shows the high-frequency period of humans’ presence on campus lying between 7 am and 8 pm. This induces a lower relative error between the fluctuating values and average value. The average daily values fed into the model for the temperature, relative humidity, wind speed and direction, with no rain observed, are illustrated in Table 1. The measured airborne pollen concentration peaks were observed in the spring season only between the 06th and 10th of April 2020. The latter clearly explains our choice of this specific 5 ays period in the year. It represents the period of the highest risk in terms of human exposure to airborne pollen on the university campus. Moreover, the technique applied to estimate the pollen production rate per tree experimentally is conducted by pollen sampling/collecting over 3 days (with only 12 h accumulated per day). Daily peaks of intermittent pollen concentration could not be predicted in the present study. This limitation can be addressed in a future study by conducting more extended periods of simulations (i.e. over a week and thus > 6 min). This is important, especially knowing that birch pollen has been repeatedly reported to be having night peaks concentrations because of long-distance transport incidents, see Kolek et al.^26^.

**Table 1:** Weather history data as mean daily values recorded in Villeneuve d’Ascq, F-59000, France at 60 masl (GPS location: 50.611208; 3.140441).

Date	T ($^{\circ }$C)	RH (%)	Wind (km/h)	Direction ($^{\circ }$)
06/04/2020	13.41	54.52	31.64	214.40
07/04/2020	14.35	57.03	5.38	109.20
08/04/2020	18.27	47.75	4.54	134.34
09/04/2020	17.00	53.33	7.40	76.20
10/04/2020	16.12	49.98	7.93	62.47

### Urban infrastructures model

**Figure 11:**
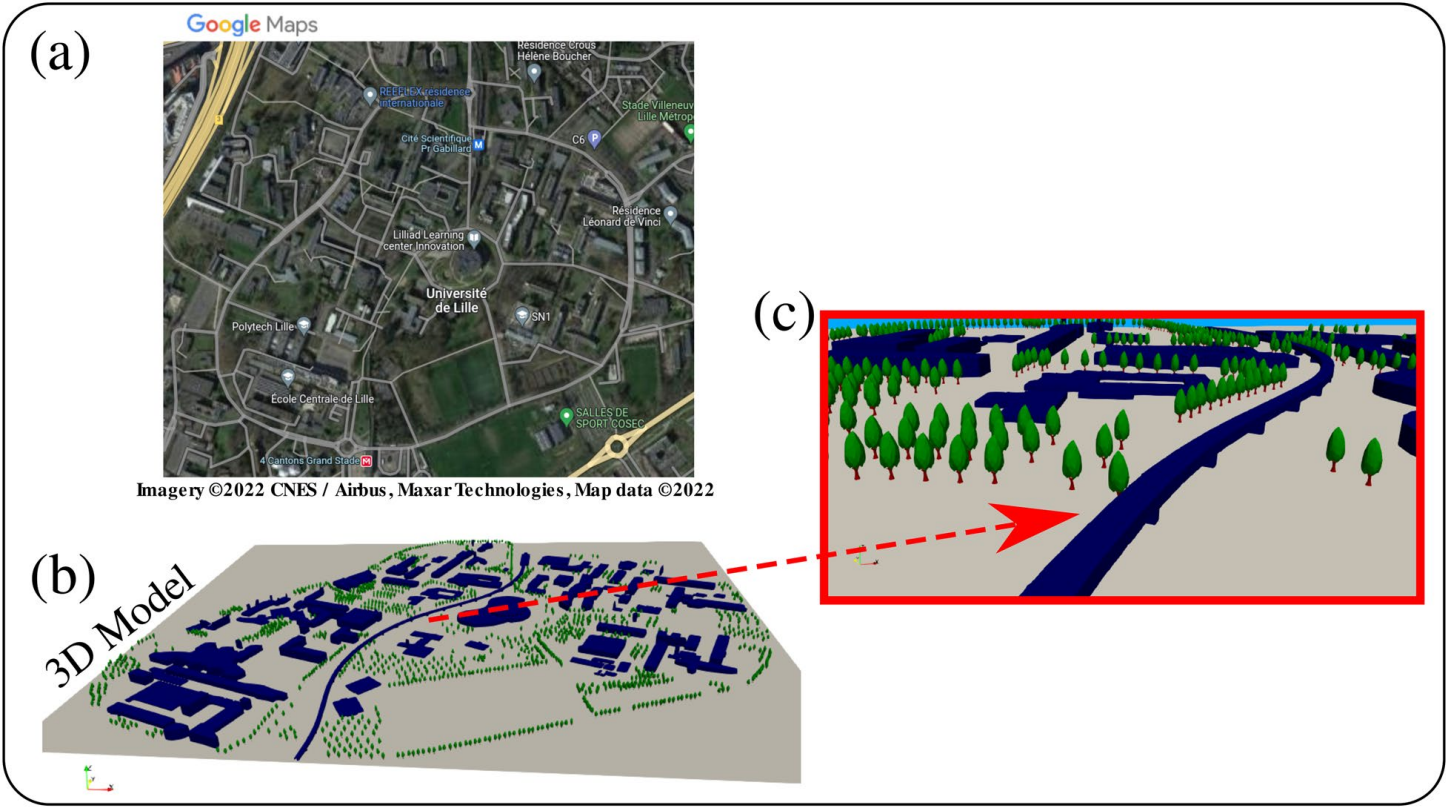
The integration of large trees into the 3D model of Fig. 9. (**a**) A green 2D map from google-maps; (**b**) constructed 3D model showing the implanted 1187 large trees; (**c**) a zoom-in perspective view showing some campus buildings, large trees and subway metro line.

Figure 11 shows a complete 3D model of the university campus in Lille (North of France) after integrating into the 3D model a representative number of the largest trees ($n_{trees}=1187$). The latter serves as an approximate major source of pollination in the model such that $n_{max}=n_{trees} \times N_{\delta \tau }$. We investigated and placed in the domain $n_{max} \approx 0.4$ million pollen grains in total at instant $t=0$ that are emitted after a period $t=\delta \tau \rightarrow ~0^+$ due to wind to flower impact (thus a natural like detachment scenario). The $n_{max} \approx 0.4$ million pollen grains corresponds to $N_{\delta \tau } \approx 320$ pollen grains per tree. We also investigated the increase of $n_{max}$ to 4 million pollen grains in total which corresponds to $N_{\delta \tau } \approx 3200$ pollen grains per tree (see Fig. 4).

### Source of pollination

We discuss below the method used to define the number of grains per tree positioned in the domain at the instant $t=0$. In our model, Fig. 4, we investigated $N_{\delta \tau }$ = 320 pollen grains (PG) per tree that corresponds to $n_{max}~=0.4 \times 10^6$ total PG in the selected domain ($n_{max}=n_{trees} \times N_{\delta \tau }$; note $n_{trees}=1187$ trees in total).

For performing the analysis, we should define the period of pollen detachment (from tree to air) $\delta \tau$, and the total surface area $S_{tot}$ for pollen production in our computational domain. For both $\delta \tau$ and $S_{tot}$, two possibilities may exist away or close to the source. For example for $S_{tot}$ it could be either:

- The total ground domain area, $S_{tot}~=~1.43~\text {km} \times 1.22~\text {km}~=~1.744 \times 10^6~ \text {m}^2$ which thus gives ($0.4 \times 10^6$ PG/$1.744 \times 10^6~\text {m}^2$ =) 0.23 PG per $\text {m}^2$ (for 6 min of simulation), or
- The total trees surface area, $S_{trees}~=~161~\text {m}^2$ per tree times 1187 trees = $1.91 \times 10^3~\text {m}^2$ which thus gives ($0.4 \times 10^6$ PG/$1.91 \times 10^3 \text {m}^2$ =) 209 PG per $\text {m}^2$ (for 6 min of simulation).

Sofiev^23^ reported that in France-Lille, there exists a total production of Birch pollen of about [0.1–3] $\times 10^9$ PG per $\text {m}^2$ per year. Converting the latter value to count for our 6 min period of simulation, we obtain [0.1–3] $\times 10^9$/($1 \times 365 \times 24 \times 60 \times 6)$ which results in [31–950] PG per m$^2$ that is relatively close but greater than 0.23 (or 209) PG per m$^2$ computed above. Therefore, we also investigated (Fig. 4) increasing $n_{max}$ or ($N_{\delta \tau }$) by a factor of 10. Increasing $n_{max}$ did have a significant impact on the overall pollen transmission paths on the campus after 5–6 min from the initial detachment of pollen grains from the trees and their dispersion in the surrounding air (see Fig. 4).

The total production of pollen can be defined as $Pr = N_{\delta \tau } / S_{tot} / \delta \tau$, i.e., which is a function of 3 physical parameters:

- $n_{max}$: the total number of pollen grains emitted by the source, e.g., tree, grass, and ground.
- $S_{tot}$: the total surface area of the sources of emission used in a computational domain, and
- $\delta \tau$: the dynamic transition time of a pollen grain from the sources, e.g., flower-borne, to airborne

Sofiev et al.^36^ developed numerical models of birch pollen emission and dispersion in the atmosphere. They linearly correlated the total number of pollen grains released from $1~\text {m}^2$ of birch forest during a year with the probability of a tree in a given grid cell and a meteorology-dependent dynamic flux correction factor.

Experimentally speaking, Jato et al.^37^ showed that a Birch tree could emit from 260 to 560 g of total pollen during the pollination season (where 80% of them are emitted during 3 days for 12 h daily^38^). Assuming an average uniform mass of 6.25 ng of a pollen grain^15^, we get an experimental production from $[0.25~\text {to}~0.56] \times 10^6$ PG/s. Here, we assumed for a tree a maximum $N_{\delta \tau }$ = 3200 PG emitted at $t=\delta \tau \rightarrow ~0^+$, thus during an infinitely short period $\delta \tau$ defined above. Unfortunately, no information exists yet in the literature on the $\delta \tau$ value, which is the period needed by the primary wind impact to detach pollen from its flower in the tree (natural detachment of pollen from wind-tree interactions). In our case, for all grains average, $\delta \tau$ is about 0.01 s, this gives us a production per tree as $N_{\delta \tau }/\delta \tau$ = $0.32 \times 10^6$ PG/s which is close to the experimental above value reported by Jato et al.^37^.

In conclusion, the above analysis shows that pollen production in the considered model is physics-based and correctly reflects previous findings^23,36^. Finally, the pollen production in the present model can be extended to account for long-distance transport of pollen^26^ and elevation effects^25^.

### Cases, tree similitude and pollen density

**Figure 12:**
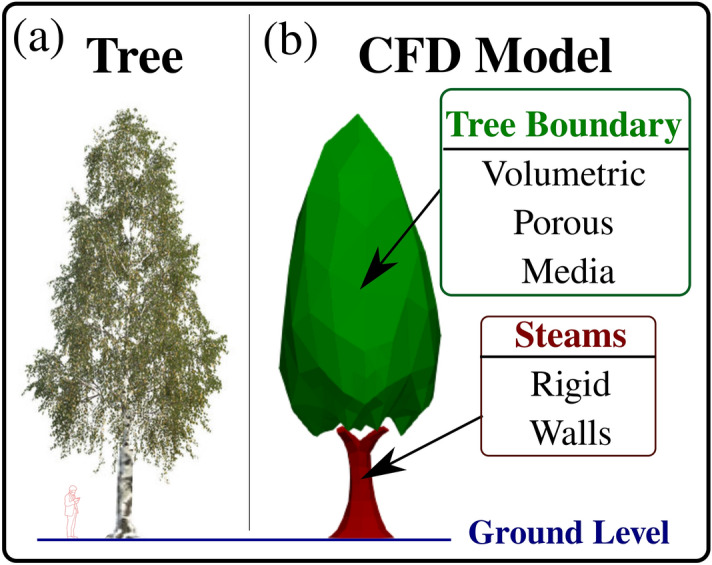
Computational modelling of a birch tree for large-scale CFD problems. (**a**) Real tree; (**b**) approximating 3D CFD model with a volumetric porous media approach applied at the leaves envelop (Darcy–Forchheimer Porous Media Model). The steams are modelled as walls with no-slip velocity boundary conditions. The ground level corresponds to $z=0$.

We applied a mean pollen density^27^ of ${\rho _p}=800~\text {kg} \times \text {m}^{-3}$ and a mean pollen grain diameter of $d_p=~20~\upmu \text {m}$ (Fig. 1). We considered the worst-case scenario for pollen dispersion around the built-in environment in the present simulations. We thus adopted a stick law at the ground and a rebound law for grains-to-surface impact. The effect of varying surface roughness properties and grain surface morphology on the stick/rebound laws is under investigation and will be presented in a future paper. The effects of the pollen grain density and total initially ejected number are also investigated by conducting two additional simulations for the weather conditions on the 6th of April 2020. Small micro-metric pollen grains are usually more dangerous to the human respiratory system and induce a higher risk of airborne pollen allergy. As shown from Fig. 12, each birch tree was modelled with a corresponding volumetric Darcy–Forchheimer porosity 3D source term model to account for the fluid flow resistance penetrating the trees under a continuum modelling approach^39^. The Darcy–Forchheimer coefficients were approximated from CFD simulations conducted by Dbouk, and Drikakis^3^ at the scale of the leaves of the tree. Five CFD simulations were conducted at five different daily weather conditions between the 6th and the 10th of April 2020.

### Computational resources

All case studies were run on an HPC cloud (High-Performance Computing) by Amazon Web Services (AWS) with multiple instances of 256 total cores. The total computational time for the 5 cases was about 7 days, thus $\approx 1.4$ days (CPU-time) per case study. This corresponds to all cases run at $n_{max}=0.4 \times 10^6$ PG, 5M cells, automatically adjusted $\Delta t$ with $\text {max}(\Delta t)=0.1$ s and at a Courant–Friedrichs–Lewy (CFL) condition CFL < 1. We ran each simulation physically for 6 min. The computational cost is prohibitive due to the computation of the dynamics of each pollen grain inside the large domain and the different stick-rebound interactions (Highly coupled Lagrangian-Eulerian CFD simulations).

## Supplementary Information

Supplementary Video 1.

Supplementary Video 2.

Supplementary Video 3.

Supplementary Video 4.

Supplementary Video 5.

Supplementary Legends.

## Data Availability

Data is available upon request from the corresponding author.
