# Supplementary material for: Risk assessment of pollen allergy in urban environments
Source: Sci Rep. 2022 Dec 6;12:21076. doi: 10.1038/s41598-022-24819-w (PMC9727162; doi:10.1038/s41598-022-24819-w)
Supplement: Supplementary file 6 — Supplementary Legends. [file 41598_2022_24819_MOESM6_ESM.pdf]

Video1
Title: Local dispersion of airborne pollen in an urban environment
Legend: Effect of weather conditions and urban infrastructure on airborne pollen aerodynamics outdoors in a university campus. Computational results shown from a top view 3D perspective for weather conditions recorded on 06 April 2020

Video2
Title: Risk assessment of pollen allergy in urban environment
Legend: Effect of weather conditions and urban infrastructure on airborne pollen aerodynamics outdoors in a university campus. Computational results shown from a top view 3D perspective for weather conditions recorded on 06 April 2020 (Top-left); 07 April 2020 (Top-right); 08 April 2020 (Bottom-left); 09 April 2020 (Bottom-right)

Video3
Title: Risk assessment of pollen allergy in a university campus
Legend: Effect of weather conditions and urban infrastructure on airborne pollen aerodynamics outdoors in a university campus. Computational results shown from a top view 3D perspective for weather conditions recorded on 06 April 2020 (Yellow-colored grains); 07 April 2020 (Red-colored grains); 08 April 2020 (Blue-colored grains); 09 April 2020 (Magenta-colored grains); 10 April 2020 (Cyan-colored grains)

Video4
Title: Pollen transport in an urban environment with large infrastructures
Legend: Pollen transport in a region R inside the university campus showing grains-trees detachment due to the wind-tree-leaves interactions. (a) t=1 sec.; (b) t=2 sec.; (c) t=3 sec. Computational case of the weather conditions on 06/04/2020

Video4
Title: Risk assessment maps obtained from large-scale computational fluid dynamics simulations
Legend: Risk assessment maps obtained from advanced large-scale computational fluid dynamics simulations, including the trees in green color (initial sources of pollen emission). Pollen allergy zones inside a university campus. The five different colours correspond to pollen grains between 0.5 and 2.5 meters above the ground level (a dangerous height interval due to the risk of humans inhaling the pollen grains). Yellow: 06/04/2020; Red: 07/04/2020; Blue: 08/04/2020; Magenta: 09/04/2020; Cyan: 10/04/2020. For the complete aerodynamics at all time steps
